# Differential regulation of two closely related integrative and conjugative elements from *Streptococcus thermophilus*

**DOI:** 10.1186/1471-2180-11-238

**Published:** 2011-10-24

**Authors:** Nicolas Carraro, Virginie Libante, Catherine Morel, Bernard Decaris, Florence Charron-Bourgoin, Pierre Leblond, Gérard Guédon

**Affiliations:** 1 Nancy-Université, UMR1128, Génétique et Microbiologie, F-54506 Vandœuvre-lès-Nancy, France; 2 INRA, UMR1128, Génétique et Microbiologie, F-54506 Vandœuvre-lès-Nancy, France

## Abstract

**Background:**

Two closely related ICEs, ICE*St1 *and ICE*St3*, have been identified in the lactic acid bacterium *Streptococcus thermophilus*. While their conjugation and recombination modules are almost identical (95% nucleotide identity) and their regulation modules related, previous work has demonstrated that transconjugants carrying ICE*St3 *were generated at rate exceeding by a 1000 factor that of ICE*St1*.

**Results:**

The functional regulation of ICE*St1 *and ICE*St3 *transcription, excision and replication were investigated under different conditions (exponential growth or stationary phase, DNA damage by exposition to mitomycin C). Analysis revealed an identical transcriptional organization of their recombination and conjugation modules (long unique transcript) whereas the transcriptional organization of their regulation modules were found to be different (two operons in ICE*St1 *but only one in ICE*St3*) and to depend on the conditions (promoter specific of stationary phase in ICE*St3*). For both elements, stationary phase and DNA damage lead to the rise of transcript levels of the conjugation-recombination and regulation modules. Whatever the growth culture conditions, excision of ICE*St1 *was found to be lower than that of ICE*St3*, which is consistent with weaker transfer frequencies. Furthermore, for both elements, excision increases in stationary phase (8.9-fold for ICE*St1 *and 1.31-fold for ICE*St3*) and is strongly enhanced by DNA damage (38-fold for ICE*St1 *and 18-fold for ICE*St3*). Although ICEs are generally not described as replicative elements, the copy number of ICE*St3 *exhibited a sharp increase (9.6-fold) after mitomycin C exposure of its harboring strain CNRZ385. This result was not observed when ICE*St3 *was introduced in a strain deriving ICE*St1 *host strain CNRZ368, deleted for this element. This finding suggests an impact of the host cell on ICE behavior.

**Conclusions:**

All together, these results suggest a novel mechanism of regulation shared by ICE*St1*, ICE*St3 *and closely related ICEs, which we identified by analysis of recently sequenced genomes of firmicutes. This is the first report of a partial shutdown of the activity of an ICE executed by a strain belonging to its primary host species. The sharp increase of ICE*St3 *copy number suggests an induction of replication; such conditional intracellular replication may be common among ICEs.

## Background

Acquisition of genomic islands (GIs) plays a key role in bacterial evolution [1,2]. *In silico *analyses revealed that numerous GIs probably belong to Integrative and Conjugative Elements (ICEs) or are ICE-deriving elements [3,4]. ICEs, including conjugative transposons, were defined as autonomous mobile elements that encode the functions needed for their excision, conjugative transfer and integration [3].

Cis-acting sequences and genes involved in a same biological process (for example conjugation) are generally grouped in a module, such as *oriT *and genes encoding relaxosome and conjugation pore. The recombination, conjugation and regulation modules are frequently grouped to form the core region of the ICEs. Although ICEs replicate during their conjugative transfer, it was originally assumed that they are incapable of autonomous intracellular replication and that their maintenance during cell growth and division only relies on their integration in the chromosome. Besides one or few core regions, they also harbor highly variable regions that encode functions potentially useful for the bacterial host [5]. Comparison of the organization of related ICEs, such as Tn*916 *and its close relatives, revealed that they evolve by deletion, acquisition and/or exchange of modules. The conjugation, tetracycline resistance and regulation modules of Tn*916 *and Tn*5397 *are closely related whereas their recombination modules are unrelated [6]. Likewise, the Tn*1549 *recombination module is closely related to the one of Tn*916*, but their conjugation and resistance modules are unrelated [7].

The closely related ICEs of the lactic acid bacterium *Streptococcus thermophilus*, ICE*St1 *and ICE*St3*, are integrated within the 3' end of the *fda *gene encoding a putative fructose 1, 6-diphosphate aldolase [8,9]. They carry recombination and conjugation modules that are almost identical (95% nucleotide identity), related regulation modules (three homologous genes showing about 85% identity; to two or three unrelated genes) and various modules that could be advantageous for their hosts (including phage resistance). Their conjugation modules are very distantly related to modules of a large group of ICEs found in firmicutes, including Tn*916 *and ICE*Bs1 *[8]. As the conjugative transfer of ICE*St1 *occurs at a frequency one thousand times lower than that of ICE*St3*, their divergent regulation modules might be involved in these very different transfer activities [10].

The activity of almost all prophages and at least some ICEs is controlled by a central repressor that can belong to two unrelated families, either cI or ImmR (also known as cI-like, although they are not homologous to cI repressor). Both types of repressor carry a HTH XRE domain that allows their binding to promoter sequences upstream from their target genes. Transfer of the element requires the inactivation of the corresponding regulator, as shown during the RecA-dependent SOS response [11-13] of many cI-encoding prophages and two ICEs, SXT from *Vibrio cholerae *[14] and ICE*Bs1 *from *Bacillus subtilis *[12], which encode respectively a cI and an ImmR repressor. Derepression of the ICE is due to the cleavage of the transcriptional regulator catalyzed by either the cI autopeptidase function [15] or a metalloprotease encoded by a gene adjacent to the gene encoding ImmR [12,16]. Previous studies showed that various stimuli can activate ICEs, such as antibiotic treatment, cell density, stationary phase, DNA damage or presence of chlorocatechol [5,11,15].

Within the regulation module of ICE*St1 *and ICE*St3*, genes encoding homologs of cI (named *arp1*) and ImmR (*arp2*) and its associated protease (*orfQ*) were identified. ICE*St1 *and ICE*St3 *are the only two characterized elements which encode both cI and ImmR repressors, suggesting a novel and complex regulatory mechanism.

In order to explain the differences of transfer frequency previously observed for ICE*St1 *and ICE*St3 *of *S. thermophilus*, a transcriptional mapping of these elements was undertaken. Furthermore their excision/replication rates were investigated in different conditions (growth medium, exponential growth, stationary phase, after exposure to DNA damaging agent). Finally the influence of the host background was also explored. These experiments revealed that the two ICEs harbor closely related core regions, differ in their transcriptional organization and regulation. They provide further evidence of ICE replication. Our results also pointed out an impact of host cell on the ICE behavior.

## Results

### Transcriptional organization and promoter analyses of the ICE*St1 *and ICE*St3 *core region

**Figure 1:**
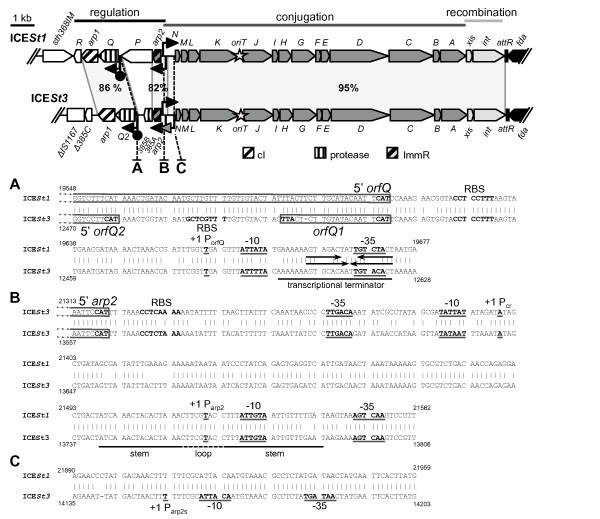
**Comparison of ICE*St1 *and ICE*St3 *regulation, conjugation and recombination modules**. Location and orientation of ORFs and a truncated IS are indicated by arrowed boxes and a rectangle, respectively. ORF names beginning with "orf" are abbreviated with the corresponding letters or numbers. The pattern of the arrowed boxes depicts the relationships of each ORF deduced from functional analyses or from BLAST comparisons. White arrowed boxes correspond to unrelated ORFs of the two elements. Black arrowed box is the chromosomal *fda *gene. The grey areas indicate closely related sequences with the nucleotide identity percentage value. The angled arrows and the lollipops indicate the experimentally demonstrated promoters and rho-independent transcription terminators predicted from *in silico *analysis (black) or unpredicted (grey). The star corresponds to the putative transfer origin. Horizontal lines delimitate functional modules with their names above. Dashed lines indicate the A, B and C intergenic regions of both ICEs; their nucleotide sequence alignments are detailed below. (**A**) Region upstream from the *orfQ *gene, (**B**) Region upstream from the *arp2 *gene, (**C**) P_arp2s _region. The position of the ribosome binding sites (RBS), initiation and stop codons are annotated in bold. Coding regions are boxed. The -10 and -35 boxes of the promoters and transcriptional start sites (+1) determined by 5'RACE PCR are in boldface and underlined. Numbers indicate the nucleotide position on the ICE sequence [GenBank:AJ278471 for ICE*St1 *and GenBank:AJ586568 for ICE*St3*]. For region upstream from the *orfQ *gene (A), arrows indicate the rho-independent transcription terminator inverted repeats. For region upstream from the *arp2 *gene (B), horizontal lines below the sequences delimitate the putative stems regions and dashed lines indicate the loop part.

Previous sequences analyses suggested that the thirteen ORFs belonging to the conjugation module and the genes encoding the excisionase and integrase (recombination module) of ICE*St1*/*3 *could be transcribed as a unique polycistronic mRNA while the regulation module could have a two-operon organization [11]. Gene organization, position of predicted promoters and rho-independent transcription terminators of the ICE*St1*/*3 *core region are schematically presented in the Figure 1. As some ICE activities were reported to be affected by growth phase and/or cell density [17,18], CNRZ368 and CNRZ385, strains carrying ICE*St1 *and ICE*St3 *respectively, were harvested in exponential growth phase as well as in stationary phase for total RNA extraction and subsequent transcriptional organization studies.

To determine which genes were co-transcribed, RT-PCR amplification of core region was performed by grouping ORFs two by two or three by three. For ICE*St1*, amplifications of *orfR/arp1/orfQ *and *orfP/arp2*, respectively, were positive while that of the *orfQ/orfP *junction was negative (see additional file 1: S1B). These data comfort the hypothesis of a two-operon organization for ICE*St1 *(see additional file 1: S1A) with a functional rho-independent transcription terminator located between the two operons. By contrast, for ICE*St3*, all the RT-PCR amplifications of the regulation module were positive (see additional file 1: S1D) indicating a co-transcription of all the regulation genes (see additional file 1: S1C). The free energy of the transcriptional terminator detected between *orf385B *and *orfQ *genes in ICE*St3 *(Figure 1) was calculated with the mFold software [19]. It is different from the one for ICE*St1 *(ΔG = -4.3 kcal.mol^-1 ^for ICE*St3 *and ΔG = -8.2 kcal.mol^-1 ^for ICE*St1*). This difference could explain why all genes of the regulation module of ICE*St3 *can be co-transcribed while two independent transcriptional units were found in ICE*St1*.

We then examined the activity of the promoter located upstream from the *orfQ *gene by Rapid Amplification of cDNA ends (5' RACE). For both elements, the start point (A nucleotide) was located seven nucleotides downstream from a -10 box separated by 17 nt from a -35 box, which overlapped the rho-independent transcription terminator (Figure 1A). This result is consistent with the *S. thermophilus *promoter consensus sequence (TTGACA - 17 nt - TATAAT) [20]. Therefore, both ICEs possess a functional P_orfQ _promoter. However, it was previously showed that ICE*St3 *differs from ICE*St1 *by a -1 frameshift in the 5' end of its *orfQ *gene (*orfQ1*) [11]. A second RBS, that could enable the translation from an initiation codon located downstream, was identified *in silico *(Figure 1A). All together, these data suggest that the *orfQ2 *gene of ICE*St3 *is truncated of 54 nucleotides at its 5' end compared to the *orfQ *gene of ICE*St1*.

**Figure 2:**
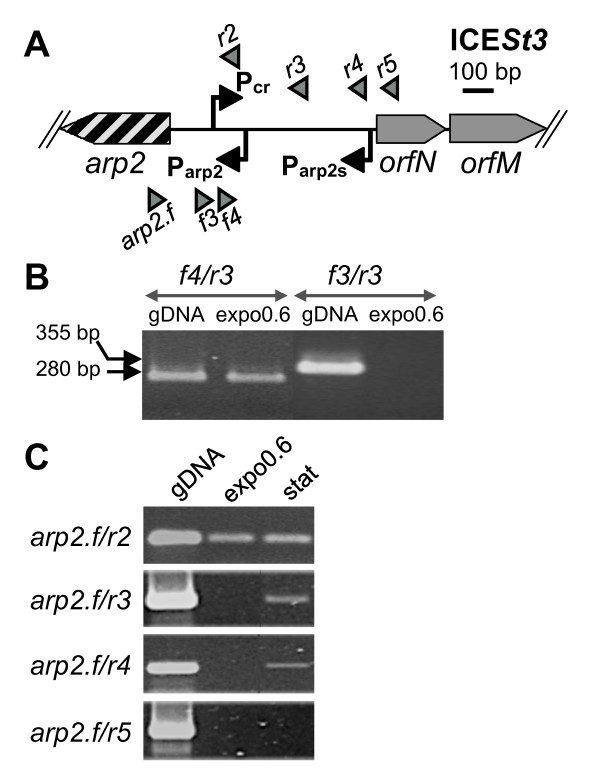
**Transcriptional analysis of the *arp2*/*orfM *region of ICE*St3***. (**A**) Schematic representation of the *arp2*/*orfM *intergenic region of ICE*St3*. Primers used for PCR analysis are represented by triangles and promoters are represented by angled arrows. (**B**) RT-PCR mapping P_cr _promoter of ICE*St3*. Amplicons are generated with primers mentioned above the gels on genomic DNA (gDNA) or cDNA synthesized from RNA extracted from cells in exponential growth phase (expo0.6). Amplicon size is given on the left. Results were identical for three independent biological replicates. (**C**) RT-PCR mapping P_arp2 _promoter of ICE*St3*. Amplicons are generated with primers mentioned on the left of the gels on genomic DNA (gDNA) or cDNA synthesized from RNA extracted from exponential growth phase (expo0.6) and stationary phase (stat) cells. The transcriptional activity upstream from the P_arp2 _promoter was detected during stationary phase. Results were identical for three independent biological replicates.

All RT-PCR amplifications targeting co-transcription of the sixteen conjugation-recombination genes of ICE*St1 *and ICE*St3 *gave amplicons (see additional file 1: S1B and S1D). Therefore, these genes are transcribed as a single polycistronic mRNA of about 14.6 kb (see additional file 1: S1A and S1C). To map more precisely the 5' end of these transcripts, other sets of primers were designed in the *arp2*/*orfN *intergenic region. For ICE*St1*, these results (data not shown) combined with 5' RACE experiments confirmed the predicted conjugation-recombination promoter, P_cr_, with a -10 box (TATAAT) located seven nucleotides upstream from the transcription start point (A) nucleotide (Figure 1B). RT-PCR experiments also localized the ICE*St3 *P_cr _promoter in the same region, between the *f4 *and *f3 *primers (Figure 2A and 2B). The ICE*St3 *precise start point could not be deduced from 5'RACE experiments because all the obtained products ended in a region located 100 bp downstream from the corresponding start point of ICE*St1*. For ICE*St1*, several 5'RACE products also ended in this region. mFold software analysis [19] revealed a conserved putative stem loop structure (ΔG = -6.7 kcal.mol^-1 ^for ICE*St1 *and ΔG = -6.4 kcal.mol^-1 ^for ICE*St3*), which could affect RNA stability. Although it could not be experimentally demonstrated, we propose, based on sequence conservation (Figure 1B), a same location of the P_cr _promoter for ICE*St3 *and ICE*St1*.

For both elements, the functionality of the predicted *arp2 *promoter P_arp2 _was established with a (A) start site located seven nucleotides downstream from a -10 box (TACAAT) (Figure 1B). For both ICEs, transcriptional analyses showed that all the promoters (P_cr_, P_orfQ _and P_arp2_), which are active during the stationary phase, are also active during exponential the growth phase (data not shown). However, an additional promoter was identified in ICE*St3 *upstream from the P_arp2 _promoter during stationary phase. Amplicons were obtained using *arp2.f*/*r3 *and *arp2.f*/*r4 *primers (Figure 2C). 5'RACE experiments revealed a start site located within a (A)_6 _stretch in this region (between the *r4 *and *r5 *primers, Figure 2C). Therefore, an alternative transcript originating from a distal *arp2 *promoter in ICE*St3 *(called "P_arp2s_") is expressed during the stationary phase (Figure 1C). This promoter does not match the classical promoter consensus as its -35 (TTATCA) and -10 (TGTAAT) boxes are separated by only 15 nucleotides (Figure 1C). The functionality of this promoter was highlighted only during stationary phase (Figure 2C) and only in ICE*St3 *(data not shown), although its sequence is strictly identical in ICE*St1 *(Figure 1C). Sequence analyses failed to detect any ORF in the 389 nucleotides between the P_arp2s _and P_arp2 _promoters.

Taken together, these data demonstrate that ICE*St1 *and ICE*St3 *do not share the same transcriptional organization of their regulation module: ICE*St1 *is organized as two operons, while in ICE*St3 *the whole module can be co-transcribed. Furthermore, ICE*St3 *possesses an additional distal promoter upstream the module, which is activated during stationary phase.

### Growth phase and MMC exposure modulate the transcription of the ICE*St1 *and ICE*St3 *core genes

Previous analyses showed a derepression of conjugative transfer of ICE*St3 *but not of ICE*St1 *after exposure to mitomycin C (MMC) [10]. In order to explain this difference, we quantified by real-time RT-PCR, three regions (*orfM*/*orfL *junction, *orfD*/*orfC *junction and integrase gene) of the conjugation-recombination transcript of ICE*St1 *and ICE*St3*.

**Figure 3:**
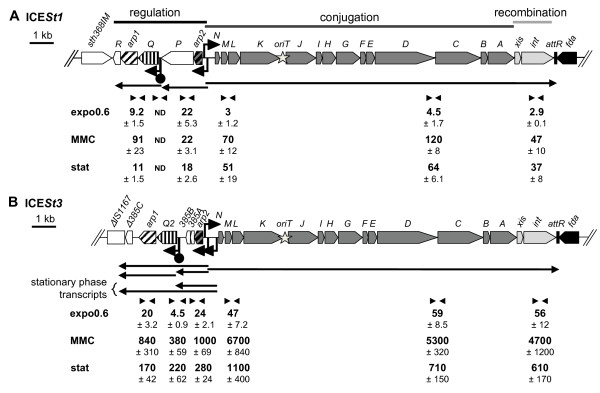
**Quantification of the transcripts of the core regions of ICE*St1 *(A) and ICE*St3 *(B)**. Arrows correspond to transcripts. Primer pairs used for cDNA quantification are represented by convergent triangles below the corresponding transcript. Other symbols used in the map are identical to those used in Figure 1. cDNA quantities determined from cells grown in LM17 medium and harvested in exponential growth phase (expo0.6) or stationary phase (stat) or after 2.5 hours of exponential growth with mitomycin C (MMC) at MIC/2 are normalized to the quantity of cDNA of *gyrA *whose transcription is constitutive [39]. Lack of amplicon is mentioned as non-detected (ND). For each condition, data are average and standard deviation from three independent biological replicates.

Quantification was done from cells harvested in exponential growth phase treated or not with MMC at the half of the minimal inhibitory concentration (MIC/2) as well as in stationary phase (Figure 3). Of note, in preliminary experiments, MMC exposure did not affect the transcriptional organization (in particular no activity of ICE*St3 *P_arp2s_), cell morphology or chain length but, as expected for a DNA damaging agent, it delayed growth, reduced DNA quantity and increased *recA *transcript levels (data not shown). Transcription of the ICE*St1 *conjugation-recombination modules was found up-regulated upon DNA damage (16-fold for the *int *gene) and in stationary phase (13-fold for the *int *gene) compared to exponential growth phase without MMC treatment (Figure 3A). The same observation was made for ICE*St3 *with a 84-fold and 11-fold increase of *int *transcript levels after MMC treatment and stationary phase, respectively (Figure 3B), indicating a probable transcriptional regulation of ICE excision. Whatever the considered region of the conjugation-recombination transcript, higher amounts were found for ICE*St3 *than for ICE*St1 *(for example, 16 to 100-fold difference in *int *gene transcript level depending on the tested condition).

For both elements, quantitative RT-PCR was also performed on three loci of the regulation module (Figure 3). In ICE*St1*, the amount of *arp2-orfP *transcripts was similar whatever the conditions considered, while the amount of *arp1 *transcripts increased 10-fold after MMC treatment (Figure 3A). Regardless of conditions, no amplification was detected at the junction between the two operons (*orfQ*/*orfP *junction), which corroborates the lack of cotranscription of these genes. For ICE*St3*, the level of *arp1 *and *orf385A*/*arp2 *transcripts increased after MMC treatment (40-fold) and in stationary phase (about 10-fold) (Figure 3B). Co-transcription of the two operons was quantified by considering the *orfQ*/*orf385B *junction. During exponential growth phase and MMC exposure, co-transcription represented 20 and 38% of transcripts respectively, indicating that the terminator and the promoter P_orfQ _were active. However, in stationary phase, the amount of this junction was similar to that of the two operons, probably reflecting an activity of the P_arp2s _promoter.

After MMC exposure during stationary phase, transcript quantities were found to be similar to the ones observed in stationary phase without MMC. Therefore, MMC has an impact on DNA metabolism (lower level of DNA) during stationary phase but does not affect levels or organization of transcripts (data not shown).

### Growth phase and mitomycin C affect ICE*St1 *and ICE*St3 *excision

**Figure 4:**
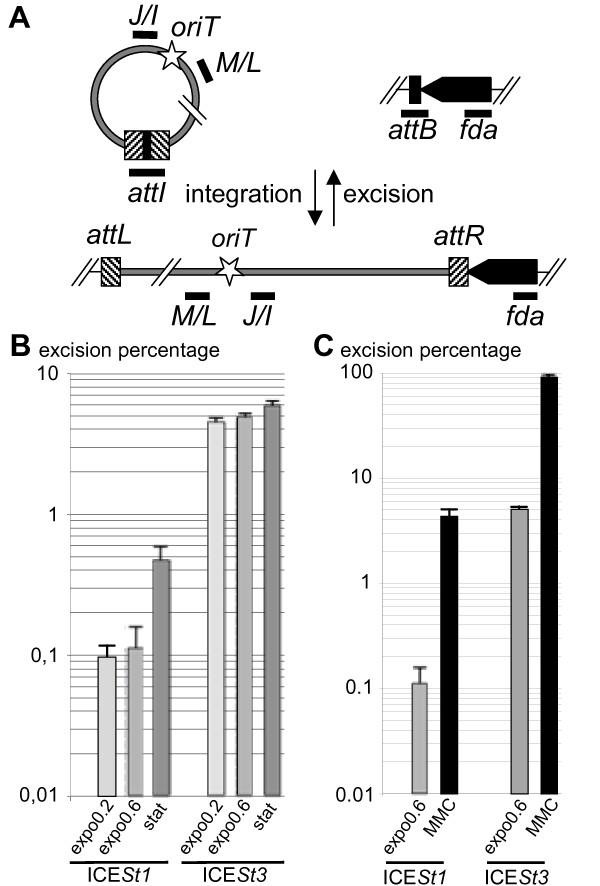
**Quantification of ICE excision**. (**A**) Localization of amplicons used for quantitative PCR. The total ICE copy number is quantified by amplification of ICE internal fragments corresponding to *orfJ*/*orfI *and *orfM*/*orfL *junctions (J/I and M/L, respectively) whereas the total chromosome number is quantified by amplification of an internal fragment of *fda*. The two products of excision, i.e circular ICE and chromosome devoid of ICE, are quantified by amplification of the recombination sites resulting from excision, *attI *and *attB *respectively. The star represents the putative transfer origin. (**B**) Effect of growth phase on excision. qPCR amplifications were performed on total DNA extracted from cells harvested during exponential growth in LM17 medium at OD_600 nm _= 0.2 (expo0.2) or OD_600 nm _= 0.6 (expo0.6) or after 1.5 hours in stationary phase (stat). (**C**) Effect of MMC treatment on excision. qPCR amplifications were performed on total DNA extracted from cells grown in LM17 medium treated or not (expo0.6) during 2.5 hours with MMC at MIC/2 and harvested at OD_600 nm _= 0.6 (MMC). Excision percentage is calculated as (*attB*/*fda*)×100. Data are presented as average and standard deviation from three independent biological replicates.

Excision is the first step of ICE transfer from host chromosome to a recipient cell, leading to a circular intermediate and an empty chromosomal integration site, *attB *(Figure 4A). The influence of the growth phase (early, mid exponential growth phase or stationary phase) and MMC treatment on ICE excision was analyzed by quantitative PCR on genomic DNA. The excision percentage was calculated as the copy number of a*ttB *sites per *fda *copy (adjacent chromosomal locus). As a control, the amount of *attB *sites was determined in strain CNRZ368ΔICE*St1 *(X. Bellanger unpublished data) and in CNRZ385ΔICE*St3 *[21] and was found equal to the amount of *fda*.

The excision percentage of ICE*St3 *was found seven-fold higher than the one of ICE*St1 *in exponential growth phase (Figure 4B), consistent with the higher level of ICE*St3 *conjugation-recombination transcript (described above), and its higher transfer frequency [10]. For both ICEs, excision frequency was higher in stationary phase compared to exponential growth phase (Figure 4B). For these experiments, cells were grown in LM17 rich medium, in which transfer has been demonstrated [10]. A similar excision rate of ICE*St3 *was measured in another rich medium (HJGL medium) that do not support the transfer of the two ICEs (data not shown). Therefore, the lack of ICE*St3 *transfer in this medium can not be due to a low excision level.

Transcriptional analyses have shown an increase of core transcript level for ICE*St3 *and ICE*St1 *after MMC treatment during exponential growth. This DNA damaging agent leads to an increase of excision percentage up to 90% for ICE*St3*, but only 4.3% for ICE*St1 *(Figure 4C). However, the increase is higher for ICE*St1 *(38-fold) compare to ICE*St3 *(18-fold). Therefore, under all tested conditions, ICE*St3 *is more active in excision than ICE*St1*.

### DNA damage induces replication of ICE*St3*

Quantitative PCR was performed to measure the amounts of excised and integrated ICEs at different growth phases and after MMC treatment. According to the previously proposed ICE model (Figure 4A) *attI *and *attB *were expected to have the same copy number after ICE excision. This was found for both ICEs whatever the tested conditions, except for ICE*St3 *DNA extracted from strain CNRZ385 exposed to MMC (with a *attI*/*attB *value of 9.95 ± 1.42). To confirm this data, the *orfM/orfL *junction localized in the conjugation module was quantified and normalized to levels of different chromosomal loci: *fda*, *dnaA *and *xerS *(data not shown). The same result was obtained with an amount of *M/L *reaching about nine-fold the one of *fda *(9.60 ± 1.04). As *fda *is adjacent to integrated ICE*St3 *and replicates prior to the ICE during host chromosome replication, ICE*St3 *could be able to replicate autonomously under this condition. Different loci along ICEs (from *J*/*I *to *M*/*L*) were quantified at similar levels (data not shown) and thus did not allow us to propose a replicative mechanism (theta *v/s *rolling-circle).

### ICE*St3 *excision and replication depend on the host strain

**Figure 5:**
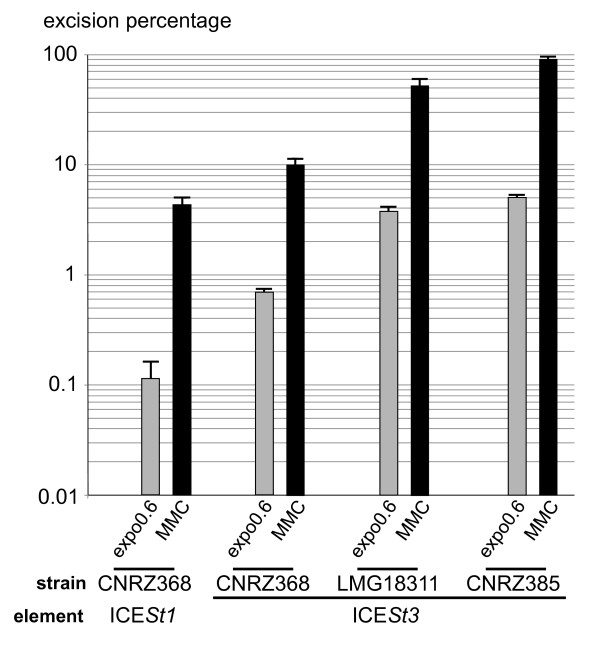
**Strain effect on ICE excision**. qPCR amplification was performed on total DNA extracted from cells harvested during exponential growth in LM17 medium at OD_600 nm _= 0.6 (expo0.6) or treated for 2.5 hours by MMC at MIC/2 and harvested at OD_600 nm _= 0.6 (MMC). ICE and host strains studied are indicated below. ICE*St3*, in strains CNRZ368 and LMG18311, was tagged with the *cat *gene, conferring chloramphenicol resistance, for transconjugant selection. To avoid ICE interference, strain CNRZ368 was previously deleted of ICE*St1 *prior ICE*St3cat *transfer. Excision percentage is calculated as (*attB*/*fda*)×100. Data are presented as average and standard deviation from three independent replicates.

To test the ICE*St3 *behavior in different *S. thermophilus *strain background, its excision percentage (*attB*/*fda*)×100 and copy number (*ML*/*fda*) were quantified. ICE*St3 *was transferred by conjugation to LMG18311, a strain initially devoid of ICE and in CNRZ368ΔICE*St1*, the strain that originally carries ICE*St1 *but has been deleted of it. ICE*St3 *excision percentage was lower in strain LMG18311 and much lower in CNRZ368ΔICE*St1 *compared to that observed in CNRZ385 strain but MMC treatment increased its excision percentage in all strains (Figure 5). In CNRZ368, excision rates of ICE*St3 *were higher than those of ICE*St1 *(Figure 5). Furthermore, the quantification showed a single copy of ICE*St3 *(1.08 ± 0.11) per chromosome even after MMC exposure (compared to 9.60 ± 1.04 copies in strain CNRZ385). This indicates a preponderant effect of the host strain on the ICE replication.

### A family of streptococcal ICEs shares related regulation and conjugation modules

**Figure 6:**
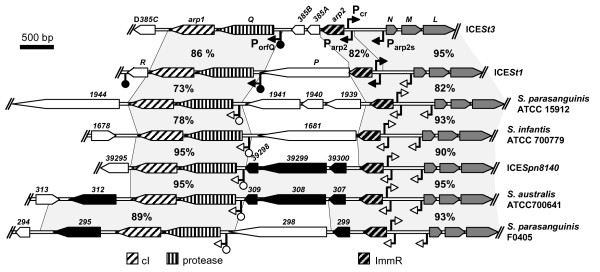
**Comparison of the conserved structure of streptococcal ICEs**. ICE names or host strain names are mentioned on the right. ORFs location and orientation of each ICE are indicated by arrowed boxes. Above, ORF names are abbreviated with the corresponding letter or number. The pattern of the arrowed boxes depicts the related ORFs, homologs to ICE*St3 *regulation and conjugation genes deduced from functional analyses or from BLAST comparisons. The grey areas indicate closely related sequences between GIs (> 70% nucleotide identity); the identity percentage between pairs of GIs is given. Homologous ORFs of unknown function and unrelated ORFs are represented by black or white arrowed boxes, respectively. The identity percentage indicated on right goes until and includes the *orfA *conjugation gene. The angled arrows and the lollipops indicate the promoters and rho-independent transcription terminators experimentally demonstrated (black) or predicted from *in silico *analysis (white). Sequences used for this analysis are from the putative ICE ICE*Spn8140 *of *S. pneumoniae *[GenBank:FR671412[22] and from the partially or completely sequenced genomes of *S. parasanguinis *ATCC15912 [GeneBank:NZ_ADVN00000000] and F0405 [GenBank:NZ_AEKM00000000], *S. infantis *ATCC 700779 [GeneBank:NZ_AEVD00000000] and *S. australis *ATCC700641 [GeneBank:NZ_AEQR00000000].

Protein and nucleic acid sequences from the regulation, conjugation and recombination modules of ICE*St1 *and ICE*St3 *were compared with sequences from firmicutes. Closely related conjugation modules (> 80% nucleotide identity all along the conjugation module) were found in the putative ICE*Spn8140 *from *S. pneumoniae *8140 [22] and in the partially or completely sequenced genomes of *S. parasanguinis *ATCC15912 and F0405, *S. infantis *ATCC 700779 and *S. australis *ATCC700641 (Figure 6). All these conjugation modules are adjacent to putative recombination modules that are unrelated or very distantly related to the ones of ICE*St1/3 *(data not shown). Nevertheless, they could be cotranscribed with the conjugation module from a P_cr _promoter similar to the one identified above since it is present at the same position as in ICE*St1*/*3 *with high sequence conservation (see additional file 2: S2A). Therefore, these conjugation-recombination modules probably belong to non identified ICEs.

All these putative elements harbor closely related regulation modules that would be transcribed divergently from the conjugation and recombination modules. All these modules possess a similar organization and encode putative cI repressors, ImmR repressors and metalloproteases related to the ones of ICE*St1/3 *(64-90% protein sequence identity) and one to four unrelated proteins (Figure 6). Sequence comparison of the intergenic core regions of the closely related streptococci ICEs revealed similar regulatory signals at the same positions as in ICE*St1*/*3 *with high sequence conservation (see additional file 2: S2B, S2C and S2D), suggesting a similar regulation.

More distantly related conjugation modules (35-70% identity for at least seven proteins with similar organization) are found not only in previously described elements - RD2 from *S. pyogenes *[23] and four elements integrated in a tRNA^Lys ^gene from four *S. agalactiae *strains [4] - but also in novel putative ICEs that we found in various *Streptococci *including *S. agalactiae *ATCC13813 (incompletely sequenced), *S. dysgalactiae *ATCC12394 (two elements), *S. downei *F0415, *Streptococcus *sp. 2_1_36FAA and *S. gallolyticus *UCN34. Only the elements found in *S. dysgalactiae *encode a putative cI repressor, ImmR repressor and metalloprotease.

## Discussion

This study of ICE*St1 *and ICE*St3*, showed that their respective transcriptional organization and their mobility behaviors differ. As previously proposed from sequence analyses, all genes included in the conjugation and recombination modules of the two elements were found to be transcriptionally linked and controlled by a single promoter. This organization allows a coordinated regulation of genes involved in conjugation and recombination, which are functionally associated during ICE transfer.

For ICE*St1 *and ICE*St3 *regulation module, the cI-like encoding gene and one to two genes located downstream are expressed from the convergent promoter P_arp2 _or from a distal conditional promoter P_arp2s_. The genes encoding metalloprotease (*orfQ*) and cI homologs belong to a different operon expressed from another promoter P_orfQ_. These two operons are separated by a rho-independent transcription terminator. The ICE*St1 *regulation module includes two independent transcriptional units. By contrast, co-transcription of all the ORFs belonging to the regulation module was observed for ICE*St3*. This is probably enabled by a weaker transcriptional terminator and perhaps a higher transcription level and the activation of the stationary phase promoter P_arp2s_. These differences probably induce ICE*St3 *and ICE*St1 *differential regulations.

The mechanisms of ICE regulation based on cI or ImmR repressors, previously described for SXT and ICE*Bs1*, are characterized by a decrease of transcript level of the cI or *immR *gene and an activation of the conjugation-recombination module transcription [5]. By contrast, in ICE*St3 *from *S. thermophilus*, a transcriptional derepression was observed for the two operons of the regulation module, whereas in ICE*St1*, only the transcript level of the operon containing *arp1 *was affected. Under all tested conditions, ICE*St3 *is more transcriptionally active than ICE*St1*. The partial derepression of transcription of the regulation module may explain the lower activation of ICE*St1 *(conjugation-recombination transcript level, excision, replication) compared to ICE*St3*. So far, ICE*St1 *and ICE*St3 *were the only known elements (ICEs and prophages) encoding homologs of both cI and ImmR repressors. The gene encoding a putative metalloprotease is generally cotranscribed and located immediately downstream from the gene encoding the ImmR repressor [12,16]. However, in ICE*St1 *and ICE*St3*, the metalloprotease gene (*orfQ*) is adjacent to the cI gene (*arp1*) but not to the cI-like gene (*arp2*), suggesting that the regulation involving both cI and cI-like regulators fundamentally differs from those identified in ICEs and related elements encoding only one regulator. Genomic analyses revealed, in various streptococci, ICEs that harbor conjugation module related to the ICE*St1*/*3 *ones These elements carry a regulation module related to the ICE*St1*/*3 *ones, suggesting that they could share a similar regulation.

After MMC treatment, the transcript levels of the recombination module increases 16-fold for ICE*St1 *and 84-fold for ICE*St3*. The 10-fold increase in ICE*St3 *copy number, after MMC treatment, could contribute to this increase of transcript levels but is not sufficient to explain its range. MMC exposure could induce an overinitiation of DNA replication with an apparent increase in origin-proximal gene expression for a short distance (≈50 kb) [24], but ICE*St1 *and ICE*St3 *are out of this area on the chromosome. MMC thus stimulates ICE transfer [10,15,25], but also increases transcription of both ICE*St3 *and ICE*St1*.

As copy number of ICE*St3 *increases after MMC treatment, the quantification of the empty chromosomal integration site underestimates the level of extrachromosomal ICEs. It is worth noticing that the increase of excision after MMC exposure does not lead to an increase of ICE*St1 *transfer. Additionally, a similar excision level was obtained for ICE*St3 *in HJGL medium, although this medium does not support ICE transfer. It shows that, besides excision, additional factors affect transfer of these elements. Similarly, although prior excision is required to observe the conjugative transfer of Tn*916*, which is an ICE that harbors a conjugation module very distantly related to the one of ICE*St1*/*3*, the transfer frequency of this ICE is not correlated with excision [26].

Some preliminary results favor the hypothesis of multiple extrachromosomal copies of ICE*St3 *(data not shown). ICEs, as their name implies, are able to excise from their host chromosome. Then the circular extrachromosomal ICE transfers to recipient cell per conjugation and simultaneously replicates by rolling-circle mechanism. The site-specific recombination leads to integration in donor and recipient chromosomes. During division, ICE transmission to the daughter cells is thought to depend on the replication and partition of the host chromosome. However, it has been recently reported that at least some ICEs can replicate independently of their conjugative transfer. In particular, the amount of excised forms of ICE*Bs1 *increases two- to five-fold under inducing conditions [27] ICE*Bs1 *replication is initiated within *oriT *and is unidirectional [27]. This replication is involved in the stability of ICE*Bs1 *and required the relaxase encoded by the element. *In silico *analysis of the putative relaxases of ICE*St1*/*3 *and of ICE*Bs1 *indicated that they are distantly related (27.4% amino acid identity for relaxase), suggesting that replication could have similar role for the two ICEs.

Furthermore, the ICE RD2 from *S. pyogenes *related to ICE*St1*/*3 *[23] and the putative ICE pKLC102 from *Pseudomonas aeruginosa *[28] were reported to be simultaneously integrated and at extrachromosomal multiple copies while pP36 from *Legionella pneumophila *is present as a multiple extrachromosomal copies in some conditions [29]. Whereas, in firmicutes, none of the known ICEs was found to encode a partitioning system; in proteobacteria, the ICEs belonging to pKLC102-ICE*clc *family encode a putative partition system [30,31].

In its host strain CNRZ368, ICE*St1 *exhibits a stable copy number, even after a stimulation of its excision and core region transcription by MMC exposure. In this strain, ICE*St3 *excision percentage is reduced 3-fold in stationary phase and nine-fold after MMC treatment and ICE*St3 *copy number is not increased compared to the one observed in the strain CNRZ385. Additional factor(s) could explain these differences (excision percentage and copy number) of ICE*St3 *in different *S. thermophilus *strains. Some host factors are likely involved in key steps of the ICE behavior, like *B. subtilis *PolC, DnaN and PcrA for ICE*Bs1 *replication [27] and IHF for SXT excision in *V. cholerae *[32]. To our knowledge, our work is the first report of partial shutdown of ICE activity by a strain belonging to the primary host species.

Analysis of recently available sequences led us to identify a set of closely related putative ICEs among various streptococcal species. All of them exhibit closely related conjugation modules but highly variable recombination modules. This suggests that these elements can transfer between various streptococcal species and exchange modules between one another. However, these regulation modules all share *arp2*, *orfQ *and *arp1 *genes (Figure 6), suggesting a fundamental function of these 3 genes in governing transfer of this ICE family. Further investigations will be required to characterize these genes and of their functional interactions with host regulators.

## Conclusions

In conclusion, the transcriptional organization of the conjugation and recombination modules of two closely related ICEs from *S. thermophilus*, ICE*St1 *and ICE*St3*, is identical, while that of their regulation module is somewhat different. Transcripts of core region and excision levels are higher for ICE*St3*, which is consistent with its higher transfer frequency. Despite these differences, the excision of both ICEs is stimulated by exposure to a DNA damaging agent and stationary phase. Data generated by the transcriptional study suggest a new mechanism of regulation of ICE*St1/3*. This behavior could be due to the atypical regulation module of these elements that encode homologues of both cI and ImmR repressors. Analyses of sequenced genomes revealed, among streptococci, a family of ICEs that encode cI and ImmR homologs and therefore could share similar regulation.

Furthermore, our results suggest that DNA damage induces not only the excision and transfer of ICE*St3 *but also its intracellular replication. This characteristic, which is not considered in the initial ICE model, may be shared by other ICEs. This study also revealed that ICE*St3 *has very different behaviors depending on its primary host species, suggesting a major role of host factor(s) in its excision and replication.

## Methods

### Strains and media

The *Escherichia coli *and *S. thermophilus *strains used are listed (Table 1). *E coli *DH5α (Gibco Life Technologies, Gaithersburg, Md, USA.) used for plasmid propagation and cloning experiments was routinely grown in LB medium at 37°C in aerobiosis [33]. *S. thermophilus *strains were grown in M17 broth (Oxoid, Dardilly, France) supplemented with 0.5% lactose (LM17) and 1% glucose (GLM17) or Hogg-Jago broth [34] supplemented with 1% glucose and 1% lactose (HJGL), at 42°C under anaerobic conditions (GENbox Anaer atmosphere generators and incubation jars from bioMérieux, Craponne, France). Agar plates were prepared by adding 2% (wt/vol) agar to the media.

**Table 1:** Strains and plasmid used in this study.

Strains or plasmids	Relevant phenotype or genotype	Reference
Strains		
*S. thermophilus*		
CNRZ368	Wild-type strain carrying ICE*St1*	INRA-CNRZ
CNRZ385	Wild-type strain carrying ICE*St3*	INRA-CNRZ
CNRZ368ΔICE*St1*	Wild-type strain cured from its ICE*St1 *resident element	X. Bellanger pers. com.
LMG18311 ICE*St3cat*	Wild-type strain carrying ICE*St3 *tagged with the *cat *gene inserted in the pseudogene *Ψorf385J*, Cm^r^	[10]
CNRZ368 ICE*St3cat*	CNRZ368ΔICE*St1 *strain carrying ICE*St3cat*, Cm^r^	This work
*E. coli*		
DH5α	*supE44 lacU169 *(φ80 *lacZ *M15) *hsdR17 endA1 gyrA96 thi-1 relA1*	[33]

Plasmid		
pSL1180	3, 4 kb, replication origin from pBR322, Amp^r^	Amersham

Abbreviations: Cm^r^, chloramphenicol resistance, Amp^r^: ampicillin resistance.

### Strain CNRZ368 ICE*St3cat *construction

To test the ICE*St3 *behavior in different *S. thermophilus *strain background, a filter mating was done as described previously [10] using the donor strain CNRZ385, carrying ICE*St3 *tagged with the *cat *gene conferring the chloramphenicol resistance [10] and the recipient strain CNRZ368ΔICE*St1*, spontaneous rifampicin and streptomycin-resistant mutant (X. Bellanger unpublished data). Triple-resistant clones were isolated and mapped for *cse *gene polymorphism [35] to confirm that they are transconjugants harboring CNRZ368 ICE*St3cat*. Three independent CNRZ368 ICE*St3cat *clones, which have similar growth parameters, mitomycin C (MMC) minimal inhibitory concentration (MIC) and *dnaA*/*xerS *rates (exponential growth phase with and without MMC treatment and stationary phase) than strains CNRZ368 and CNRZ368 cured of ICE*St1 *were used for each experiments.

### Growth conditions

*S. thermophilus *strains were grown at 42°C in 30 mL of LM17 medium to an optical density at 600 nm of about 0.7. Measures of OD_600 nm _were performed with the Genesys 20 spectrophotometer (Thermo scientific, Illkirch, France). Cells were diluted until OD_600 nm _= 0.05 into 50 mL of preheated medium (42°C) and harvested at early (OD_600 nm _= 0.2), mid exponential growth phase (OD_600 nm _= 0.6) or stationary phase (after 1.5 hours at OD_600 nm _= 1.5) with or without MMC exposure during 2.5 hours at the half of the minimal inhibitory concentration (MIC/2 = 0.1 μg/mL, for all the *S. thermophilus *strains used in this study) for genomic DNA or RNA extractions. Cultures were centrifuged at 13, 000 g during 15 min at 42°C and cell pellets were stored at -80°C.

### DNA manipulation

DNA quantity along the MMC exposure was investigated by colorimetric DNA dosage [36]. Genomic DNA of *S. thermophilus *was extracted as described previously [37]. Plasmid DNA isolation was performed using Genelute Plasmid Miniprep Kit (Sigma-Aldrich, Lyon, France). DNA fragment recovery was performed using the High Pure PCR Product purification kit (Roche, Neuilly-sur-Seine, France). DNA cloning, ligation and restriction enzyme digestion were all carried out according to standard procedures [33] or according to specific recommendations of the supplier (New England Biolabs, Evry, France). PCR primers were designed with the PrimerQuest software http://www.idtdna.com/scitools/applications/primerquest/ and synthesized by Eurogentec (Angers, France) at 100 μM. PCR and high fidelity PCR were carried out according to the instructions of the ThermoPol PCR kit (New England Biolabs, Evry, France) and of the Triple Master PCR System (Eppendorf, Le Pecq, France), respectively. Sequencing reactions on RACE PCR amplifications were performed by Cogenics (Beckman Coulter genomics, Villepinte, France).

### Reverse transcription PCR (RT-PCR)

Cell pellets were resuspended in 1 mL of Kirby mix (1% w/v of N-Lauroylsarcosine, 6% w/v p-aminosalicylic acid sodium salt, 0.1 M Tris HCl pH = 8, 6% v/v phenol pH = 8). Then total RNAs were extracted as described previously [38]. The cDNAs were obtained by reverse transcription of 1 μg of DNase I-treated (Euromedex, Souffelweyersheim, France) total RNA with M-MLV reverse transcriptase (Invitrogen, Villebon sur Yvette, France) and random hexamer primers (Applied Biosystems, Villebon sur Yvette, France). PCR amplification of *gyrA *(40 cycles) was performed using *gyrAR1 *and *gyrAR2 *primers (see additional file 3: table S1) on retrotranscribed RNA and non retrotranscribed RNA, and used as positive and negative control, respectively. The quality of generated cDNA was controlled by amplifying a 1000-bp fragment by the *J/I.f *and *G/H.r *primers (see additional file 3: table S1). Transcriptional mapping was done using primers amplifying less than 1000-bp with a standard PCR program: 30 s at 95°C for denaturation, annealing 30 s at 50°C and extension 1 min at 72°C for 30 cycles. Primers are listed in the additional file 3, table S1 in part and available upon request for the rest.

### Mapping of 5' extremity of RNA

5' ends of transcripts were mapped by Rapid Amplification of cDNA Ends using the 5'RACE PCR kit (Invitrogen, Villebon sur Yvette, France). PCR products were directly sequenced to determine the 5' ends. When they can not be precisely determined by direct sequencing, PCR products were subsequently cloned in pSL1180 (Table 1); 15 and 12 clones were sequenced for ICE*St1 *and ICE*St3 *respectively. Primers used are listed in the additional file 3 table S1.

### Quantitative PCR

Quantitative PCR (qPCR) was performed with 2 fg-200 ng DNA or cDNA, 5 μL qPCR Mastermix (Bio-rad, Marnes-la-Coquette, France) and 450 pM primers (see additional file 3: table S1) in 10 μL final volume. After activation of the hot start polymerase (30 s at 98°C), 40 cycles were performed: denaturation 10 s at 95°C and annealing/extension 45 s at 50°C for cDNA or denaturation 30 s at 95°C, annealing 30 s at 50°C and extension 1 min at 72°C for gDNA. The melting curve of the PCR product was analyzed with CFX manager software (Bio-rad, Marnes-la-Coquette, France) to verify PCR specificity. It was acquired each 0.5°C for 1 s by heating the PCR product from 60°C to 95°C. For each run, a standard dilution of the DNA fragment (preliminary obtained by PCR) was used to check the relative efficiency and quality of primers. A negative control (ultra-pure water obtained by the Direct8 Milli-Q system, Millipore, Molsheim, France) was included in all assays. Each reaction was performed at least in duplicate. Real-time PCR was carried out on a C1000 Thermocycler coupled by a CFX96 real-time PCR detection system (Bio-Rad, Marnes-la-Coquette, France). Strains depleted for their resident ICE, CNRZ368ΔICE*St1 *(X. Bellanger unpublished data) and CNRZ385ΔICE*St3 *[21], which have equal amount of *attB *and *fda*, were used as controls. cDNA quantities of studied genes were normalized to the amount of cDNA of the *gyrA *gene, whose transcription is considered as constitutive [39]. Similar results were obtained when the *ldh *gene, encoding the lactate dehydrogenase, was used for normalization [40]. Data are expressed as mean ± SD. Statistical analysis was performed with Student's E test. A *p *value < 0.05 was considered statistically different.

### Sequence analysis

Protein and nucleic acid sequences from the recombination, regulation and conjugation modules of ICE*St1 *and ICE*St3 *were compared with sequences from Firmicutes on the NCBI server http://www.ncbi.nlm.nih.gov using BLASTP, BLASTN and/or tBLASTN. Identified sequences are from ICE*Spn8140 *of *S. pneumoniae *[GenBank:FR671412[22]] and from the partially or completely sequenced genomes of *S. parasanguinis *F0405 [GenBank:NZ_AEKM00000000] and ATCC15912 [GeneBank:NZ_ADVN00000000], *S. australis *ATCC700641 [GeneBank:NZ_AEQR00000000] *S. infantis *ATCC700779 [GeneBank:NZ_AEVD00000000], *S. agalactiae *ATCC13813 [GenBank:AEQQ01000089], *S. dysgalactiae *ATCC12394 [GenBank:CP002215], *S. downei *F0415 [GenBank:NZ_AEKN01000010], *Streptococcus *sp. 2_1_36FAA [GenBank:NZ_GG704942] and *S. gallolyticus *UCN34 [GenBank:NC_013798].

## Authors' contributions

Conceived and designed the experiments: NC VL FCB PL GG. Performed the experiments: NC VL CM. Analyzed the data: NC VL FCB PL BD GG. Wrote the paper: NC VL FCB GG. All authors read and approved the final manuscript.

## Supplementary Material

Additional file 1 **Fig. S1: Determination of transcriptional units of the ICE core region in stationary phase**. ICE*St1 *(**A, B**) and ICE*St3 *(**C, D**). For (A) and (B), location and orientation of ORFs and a truncated IS are indicated by arrowed boxes and rectangle, respectively. Above, ORF names beginning with "orf" are abbreviated with the corresponding letter or number. The pattern of the arrowed boxes depicts the putative function and/or relationships of each ORF deduced from functional analyses or from BLAST comparisons. White arrowed boxes correspond to unrelated ORFs of the two elements. Black arrowed box is the chromosomal *fda *gene. Star represents the putative origin of transfer. Horizontal lines delimitate functional modules with their names above. Arrows below each ICE represent transcripts deduced from the results given in B and D. For (B) and (D), RT-PCR amplification was used to determine if RNA spans the ORF end and the beginning of the following or next ORF. For each amplifications, the positive control performed on genomic DNA is presented on the left and the amplification obtained on cDNA is showed on the right. ORFs named above indicate the examined region and numbers below indicate the calculated amplicon size. Similar results were generated with RNA from three independent biological replicates and cells in exponential growth phase. A PCR was performed without reverse transcriptase step, in order to control for the absence of DNA contamination (not shown). Click here for file

Additional file 2 **Fig. S2: Multiple alignment of the four promoter regions of the seven closely related streptococcal ICEs**. (**A**) P_orfQ_, (**B**) P_cr_, (**C**) P_arp2 _and (**D**). P_arp2s_. Spara_15912, *S. parasanguinis *ATCC15912; Sinf_700779, *S. infantis *ATCC 700779; ICE*Spn8140 *from *S. pneumoniae *8140; Saus_700641, *S. australis *ATCC700641; Spara_F0405, *S. parasanguinis *F0405. The -10 and -35 boxes of the promoters are grey coloured and the transcriptional start sites (+1) are in boldface. For P_orfQ _region (A), the change in free energy (ΔG) of the underlined terminator is indicated on the right. For P_arp2 _region (C), horizontal lines below the sequences delimitate the putative stems regions and dashed lines the loop parts, which might be involved in mRNA cleavage. Click here for file

Additional file 3 **Table S1**. Main primers used in this study. Click here for file
