# Supplementary figures and images for: Differential regulation of two closely related integrative and conjugative elements from Streptococcus thermophilus
Source: BMC Microbiol. 2011 Oct 24;11:238. doi: 10.1186/1471-2180-11-238 (PMC3234194; doi:10.1186/1471-2180-11-238)

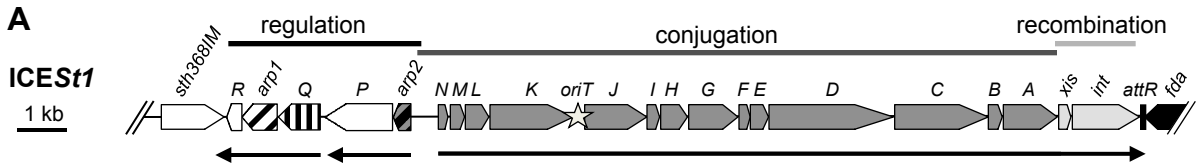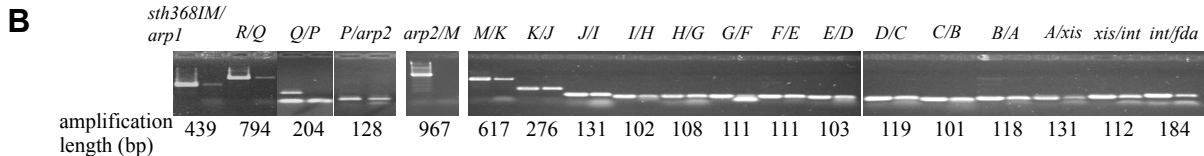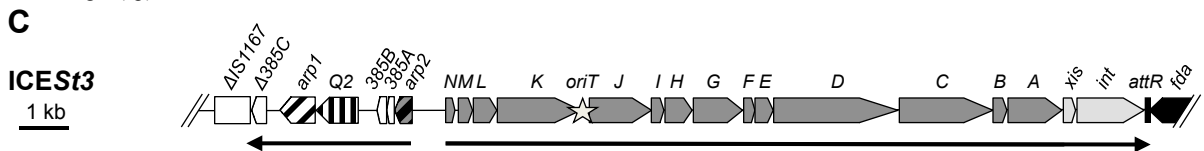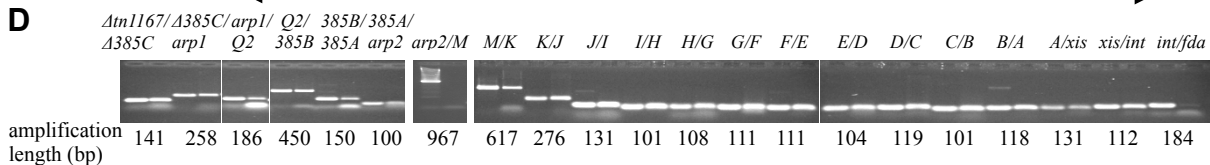

Supplement: Additional file 1 — Fig. S1: Determination of transcriptional units of the ICE core region in stationary phase. ICESt1 (A, B) and ICESt3 (C, D). For (A) and (B), location and orientation of ORFs and a truncated IS are indicated by arrowed boxes and rectangle, respectively. Above, ORF names beginning with "orf" are abbreviated with the corresponding letter or number. The pattern of the arrowed boxes depicts the putative function and/or relationships of each ORF deduced from functional analyses or from BLAST comparisons. White arrowed boxes correspond to unrelated ORFs of the two elements. Black arrowed box is the chromosomal fda gene. Star represents the putative origin of transfer. Horizontal lines delimitate functional modules with their names above. Arrows below each ICE represent transcripts deduced from the results given in B and D. For (B) and (D), RT-PCR amplification was used to determine if RNA spans the ORF end and the beginning of the following or next ORF. For each amplifications, the positive control performed on genomic DNA is presented on the left and the amplification obtained on cDNA is showed on the right. ORFs named above indicate the examined region and numbers below indicate the calculated amplicon size. Similar results were generated with RNA from three independent biological replicates and cells in exponential growth phase. A PCR was performed without reverse transcriptase step, in order to control for the absence of DNA contamination (not shown). [file 1471-2180-11-238-S1.PDF]
