# Supplementary material for: Differential regulation of two closely related integrative and conjugative elements from Streptococcus thermophilus
Source: BMC Microbiol. 2011 Oct 24;11:238. doi: 10.1186/1471-2180-11-238 (PMC3234194; doi:10.1186/1471-2180-11-238)
Supplement: Additional file 2 — Fig. S2: Multiple alignment of the four promoter regions of the seven closely related streptococcal ICEs. (A) PorfQ, (B) Pcr, (C) Parp2 and (D). Parp2s. Spara_15912, S. parasanguinis ATCC15912; Sinf_700779, S. infantis ATCC 700779; ICESpn8140 from S. pneumoniae 8140; Saus_700641, S. australis ATCC700641; Spara_F0405, S. parasanguinis F0405. The -10 and -35 boxes of the promoters are grey coloured and the transcriptional start sites (+1) are in boldface. For PorfQ region (A), the change in free energy (ΔG) of the underlined terminator is indicated on the right. For Parp2 region (C), horizontal lines below the sequences delimitate the putative stems regions and dashed lines the loop parts, which might be involved in mRNA cleavage. [file 1471-2180-11-238-S2.PDF]

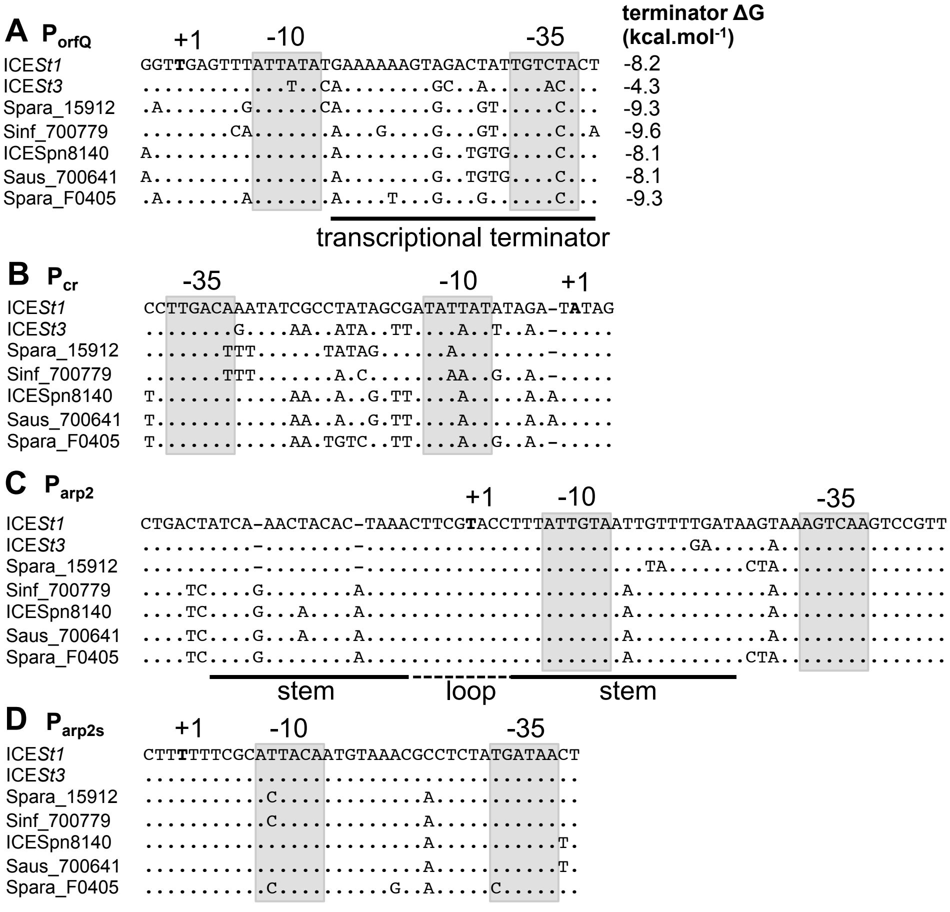

A P_orfQ
+1
-10
-35
terminator ΔG (kcal.mol-1)
ICESt1 GGTTGAGTTTATTATATGAAAAAGTAGACTATTGTCTACT -8.2
ICESt3 -4.3
Spara_15912 -9.3
Sinf_700779 -9.6
ICESpn8140 -8.1
Saus_700641 -8.1
Spara_F0405 -9.3
transcriptional terminator
B P_cr
-35
-10
+1
ICESt1 CCTTGACAAATATCGCCTATAGCGATATTATATAGA-TATAG
ICESt3
Spara_15912
Sinf_700779
ICESpn8140
Saus_700641
Spara_F0405
C P_arp2
+1
-10
-35
ICESt1 CTGACTATCA-AACTACAC-TAAACTTCGTACCTTTATTGTAATTGTTTTGATAAGTAAAGTCAAGTCCGTT
ICESt3
Spara_15912
Sinf_700779
ICESpn8140
Saus_700641
Spara_F0405
stem
loop
stem
D P_arp2s
+1
-10
-35
ICESt1 CTTTTTTCGCATTACAATGTAAACGCCTCTATGATAACT
ICESt3
Spara_15912
Sinf_700779
ICESpn8140
Saus_700641
Spara_F0405
