# Supplementary material for: Differential regulation of two closely related integrative and conjugative elements from Streptococcus thermophilus
Source: BMC Microbiol. 2011 Oct 24;11:238. doi: 10.1186/1471-2180-11-238 (PMC3234194; doi:10.1186/1471-2180-11-238)
Supplement: Additional file 3 — Table S1. Main primers used in this study. [file 1471-2180-11-238-S3.PDF]

Name	Sequence (5'→ 3')	Use
01.arp2.f	TGTCAGGTGAGCTTGTTT	Transcriptional analysis of the *arp2/orfM* intergenic région of ICE*St1* and ICE*St3*
03.arp2.f	GCCACTTCAACTTGAGTAAA	
f1	GACTACCTAAAGAGATTGGCCG	
f2	CTGACAACCAGAGGACTGACTATC	
01.f3	CCCTTGACAAATATCGCCTATAGC	
03.f3	CCCTTGACAGATATAACCATAAGT	
03.f4	CTATCAGAGTGAGATCATTGACA	
01.r1	GCTATAGGCGATATTTGTCAAGGG	
03.r1	ACTTATGGTTATATCTGTCAAGGG	
r2	GATAGTCAGTCCTCTGGTTGTCAG	
r3	CGGCCAATCTCTTTAGGTAGTC	
03.r4	AGTAGCTTGAGGGTAAGTCTATCT	
03.r5	AGCTTGTAGTTTGGGCGAGTAG	
orfM.r	CATAGTAAGCAGGGCCGATAAAG	
01.PorfQARN	GACATTGGCTTCTCGTTCTAGCAA	Detection of the transcriptional start point associated to P_{orfQ}
01.PorfQADN	CCCGTCGACTAGATTCTGCGAAATAGTTCCCATCAT	
03.PorfQARN	GGACAATATCTGGCATCAATACC	
03.PorfQADN	CCCGTCGACCCACAGCAGAAAAAATATTCGC	
01.PorfMARN	GTAGCTTGAGAGTAAGTCCATCTATAT	Detection of the transcriptional start point associated to P_{arp2}
01.PorfMADN	CCCGTCGACATTCCTCTTTCCACGGTGGCAACGTTTA	
03.PorfMARN	TTGTGCAATCCTGACTAGATTATCT	
03.PorfMADN	CCCGTCGACCTTGTGTTGGTTTTTTGACTCCA	
01.Parp2ARN	CTTCTCTTCATCACTCAATCCCTT	Detection of the transcriptional start point associated to P_{orfM}
01.Parp2ADN	CCCGTCGACAGTTCAATGTTGTCCAACTCGTCGTT	
03.Parp2ARN	GATCCCTCTTTCCACGGTGGCAA	
03.Parp2ADN	CCCGTCGACAATTACCATCGCTTGCCCGGCCA	
03.Parp2sARN	TCCGTTTATGGTTGAAGTGAAGATA	Detection of the transcriptional start point associated to P_{arp2s}
03.Parp2sADN	CCCGTCGACAAATAAGTATTTATGGGCTTCCACGC	
01.orfR/arp1.f	GTAATCAATTTGTAAATCACCTGCG	Quantification of the transcript belonging to the regulation module and containing *arp1*
01.orfR/arp1.r	TGATGAAGATCCACGAGTAGTAGG	
03.op1.1.f	AGGTCTGAGAGTTCCACACTAC	
03.op1.1.r	CCTCATGGGTTTCAGGAGATTC	
01.op2.1.f	GAATGATCTGCGAGCTGGAT	Quantification of the transcript belonging to the regulation module and containing *arp2*
01.op2.1.r	GTTGGGTGACAAGGCAACTTAC	
03.op2.1.f	CACATCATACCTTGTTCCCATCG	
03.op2.1.r	GAGAGTTGAACGTGGTCTAACA	

M/L.f	AGCTCTCAATGTCTTTGCGGTT	Transcript quantification of *orfM/orfL* junction belonging to the conjugation module of ICE*St1* and ICE*St3*
M/L.r	ATGACACGATAGGTCTTGTCCG	
J/I.f	GGAGGCTATCTTAAGACTCCTCAAA	Transcript quantification of *orfJ/orfI* junction belonging to the conjugation module of ICE*St1* and ICE*St3*
J/I.r	AAGGTACTTAGTCCACCTGACC	
D/C.f	AGCAACAGTATCTTCAAGTCTCG	Transcript quantification of *orfD/orfC* junction belonging to the conjugation module of ICE*St1* and ICE*St3*
D/C.r	GGTTCCATGTAGGACTTTAGGGAG	
qPCRmodrec.f	CTAACCGGAATGCGTATTGGTG	Transcript quantification of *int* belonging to the recombination module of ICE*St1* and ICE*St3*
qPCRmodrec.r	CTATCTTCTGAGGCTCCTCCAT	
gyrAR1	GATGCCGTTAAATTGATGAT	Transcript quantification of the housekeeping *gyrA* of CNRZ368 and CNRZ385
gyrAR2	GAGCCTTTACCAGTTTCGTA	
ldh.F	AAGCTATCCTTGACGATGAA	Transcript quantification of the housekeeping gene *ldh* of CNRZ368 and CNRZ385 [40]
ldh.R	AATAGCAGGTTGACCGATAA	
fda.f	GTGGATGTTACCGATACCTG	Integration region quantification for normalization of excision and replication
fda.r	CTATCGGTGGTGAAGAAGAC	
dnaA.f	CCCGAGTTGCTATTCTTACC	Origin of replication of *S. thermophilus* chromosome quantification
dnaA.r	GTGATCGTATCTAACTGGCG	
xerS.f	GACGTTCACGTAGATAGAGGAC	Terminus of replication of *S. thermophilus* chromosome quantification
xerS.r	GGTACCGTATTCTTTCACGACC	
131.2	TGTTGCTGAATACGAAGC	Quantification of *attB* site resulting from ICE excision
132.3	GGACTACTAAGAGAACAT	
